# Effect of Distortion in Velocity Profile on Flow Measurements Using Averaging Flow Sensors

**DOI:** 10.3390/s20102839

**Published:** 2020-05-16

**Authors:** Mirosław Kabaciński, Janusz Pospolita

**Affiliations:** Mechanical Department, Chair of Thermal Engineering and Industrial Facilities, Opole University of Technology, 45-271 Opole, Poland; j.pospolita@po.edu.pl

**Keywords:** flow-averaging pitot tubes, elbow flow, CFD, flow-behind obstacles

## Abstract

This article reports the analysis on the flow interference due to the pressure-averaging probe. The disturbance in the velocity field generated by elbows with radii of curvature of 1D and 3D (D-internal diameter of a pipeline) were investigated based on the results recorded by selected probes with various cross-sections installed at distances in the range of 3D to 20D from the elbow. The uncertainty in the current measurement is 8.26% for a circular probe. The uncertainty was related to the distance between the probe and the elbow and the relative location of the elbow with respect to the probe. This uncertainty was found to significantly depend on the design of the cross-section of the probe. Unsteady RANS computations with the standard k-ω model are used in the current numerical approach. The distributions of the velocities and pressures of fluid flow in the vicinity of the probe were determined. Mathematical modeling can also provide guidance to improve and adapt the metrological properties of the flowmeter to the conditions of its place of installation.

## 1. Introduction

The measurements of the flow rates and volume fractions of phases are some of the most common procedures applied in industry. Such measurements often face a number of impediments, the effects of which are reflected in increased measurement uncertainty. The reasons for such difficulties are varied. For instance, gas can contain dust particles or liquid droplets. In general, the occurrence of a second phase in flow results in metrological problems. In such cases, standard orifices are often applied to determine the additional measurement uncertainty, as described in Ref [1]. Ref [2] presents a method of measuring a two-phase gas–liquid stream with a large part of the gas phase. Two slotted orifices were used to measure the pressure difference on each of them. A mathematical model of measurement was formulated. Gas and liquid phase streams were determined on-line based on the pressure difference signals and the developed model. The neural network method was used to findings the model parameters. In this field, studies have been conducted on the effects of the presence of liquid droplets on the measurements performed by applying the flow-averaging Pitot tubes proposed in [3]. Another impediment that can arise in such measurements is related to the fast and considerable fluctuations in the physical parameters of fluids as well as the occurrence of transient states, such as pulsations accompanying the flow as described in Ref [4]. Another potentially challenging task in terms of flow metrology is associated with the need to measure a considerable range of flow rates.

Some of the most common installation-related and metrological problems in engineering are associated with the short runs of straight tubes upstream and downstream of a flowmeter in a pipeline. This is because the maintenance of adequate sections of straight pipes is not always feasible. Such problems are particularly common for channels with large diameters installed as part of a boiler apparatus. This is only a single case when the design of an apparatus, overhaul requirements and access routes determine the length of the straight piping sections in relation to the location of a flowmeter. In general, the occurrence of such conditions in an installation leads to an additional measurement uncertainty, the exact value of which is difficult to generally specify. A wealth of information is available regarding the influence of disturbance in the velocity profile on the systematic error in measurements only with regard to the case of the use of a standard orifice. The standard shown in Ref [5] contains data for the length of the required straight sections of tubes downstream of selected obstructions depending on the type of the flowmeter and their geometry, coupled with the systematic measurement error relative to the flow coefficient C given with regard to the standard orifice. Systematic errors in ultrasonic, electromagnetic and Venturi flowmeters installed in a double-elbow system have been described in Ref [6]. For other types of flowmeters, this information is scarce and inconsistent.

V-cone flowmeters are the ones with relatively low-sensitive flow disturbance. They are also characterized by low measurement error, no moving parts, durability and resistance to high temperatures. Hence, the interest in this type of flowmeter designs and a significant number of tests in this area. In Ref [7] the effect of cone vortex angle on the value of the flowmeter discharge coefficient was examined. A relatively small decrease in C_d_ value was found with the increase in cone angle. Numerical tests were also used to analyze the impact of stream swirl on the measuring pressure difference. It was found that as the swirl angle increases, the flow factor increases. In the range of tested angles 10°, 20° and 30° C_d_ values were 0.749, 0.753 and 0.762 with C_d_ value 0.738, when there is no swirl. In Ref [8] the numerical tests of the influence of elbows located x/D = 4 in front of the V-cone flowmeter on its indications are presented. The effect of one or two elbows lying in one or two perpendicular planes were examined. It was found that in the analyzed configurations of the flow system the influence is within ±2%.

Research has also been undertaken into the possibility of using a V-cone flowmeter for measuring two-phase flows. The study in Ref [9] presents the tests on the flow measurements of gas–liquid mixture with a high concentration of gas. A measurement method was proposed that allows on the basis of developed correlations of measured pressure differences to estimate on line the gas and liquid flow. In Ref [10] the flow of an air–water mixture in a horizontal pipe with an installed V-cone flowmeter was tested experimentally and numerically. Visualization in experimental research and the numerical simulations allowed the analysis of the structure of two-phase flow in the environment of the flowmeter, distribution of both phases and the impact of the liquid phase on the pressure values in the vicinity of the flowmeter.

For the case where one of the phases, such as gas (whose flow rate is to be measured), has a high temperature in the range of several hundred degrees, and the diameter of the pipeline is D > 0.5 m, the range of accessible flowmeters with potential applications is small. An effective solution is associated with the use of flow-averaging Pitot tubes. Owing to the negligible loss in permanent pressure and easy installation, such flowmeters have an increasing range of applications. They are likely to offer a substitute to the use of a standard orifice in all applications where they are technically feasible and economical. In particular, this applies to the Venturi tube flowmeters used in channels with large diameters, as presented in Ref [5]. Moreover, with regard to the flow-averaging Pitot tubes, there is a scarcity of information regarding the impact of their installation based on metrological properties. Ref [11] focused on the variation in the flow coefficient due to the installation of a probe with a circular cross-section installed at distances of 1D, 11D and 21D (D–internal diameter of a pipeline) downstream of the pipe elbow, with two elbows in one and two planes and for two locations of the probes. Ref [12] constitutes an extension to the scope of studies with regard to other probe types, located in various planes and at various distances from the flow obstacle. It was shown that in addition to the distance from the obstacle, it is necessary to consider the orientation of the plane in which the probe is installed. The results show that this systematic error does not decrease linearly with increasing distance between the probe and the obstruction. A manual in Ref [13] describes a summary of information regarding the impact of various flow obstructions on additional error in the flow measurements due to the metering process. A lack of the data on the type of the applied probe and its orientation plane leads to studies offering merely a degree of qualitative assessment. The data found there show that in case of parts, such as valves and elements, causing flow turbulence, their installation, even at a distance of 30 to 40 tube diameters from the probe, can result in flow distortion.

Ref [14] contains a proposition involving criterion-based coefficients to be applied to assess the development in the velocity profile upstream of a flowmeter. The values of such coefficients several lengths away from the elbow were successfully determined. In this study, the suitability of the established criteria was proved to apply to the validation of numerical simulations. However, attempts at correlating the values of these parameters with the systematic measurement error caused by the installation of flowmeters (conducted by the authors of this study) did not yield satisfactory results.

This study contains an assessment of additional measurement error resulting from the installation of flowmeters examined downstream of elbows with various radii of curvature. The study involved three flowmeters differing in terms of the cross-section of the averaging probes. Numerical modeling was applied for a physical interpretation of the selected examples. The velocity and pressure fields determined in flow across a streamlined probe downstream of an obstruction also provide guidance for the design requisite of a flowmeter and details of its location, where this can help reduce the additional measurement error resulting from flow disturbance.

## 2. Materials and Methods

### 2.1. Flow-Averaging Pitot Tube Flowmeters

**Figure 1:**
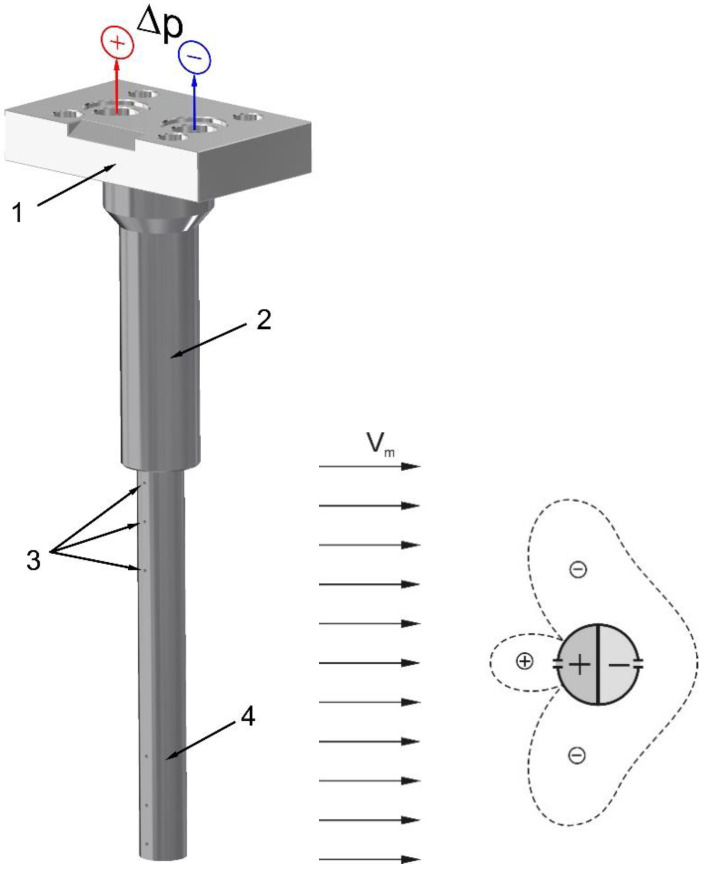
Averaging Pitot tube: 1-head, 2-cylindrical part (mounted in choke), 3—impulse holes, 4—flow sensor.

The basic component of the examined flowmeters was the flow-averaging Pitot tube (Figure 1), which comprises bore holes to receive the total pressure and chambers in which the pressure is averaged.

The measured differential pressure Δ*p* = *p*^+^ − *p*^−^ in the chambers gives the volumetric flow rate in accordance with

(1) $$q_{v}=K_{\infty }A\sqrt{\frac{2\Delta p}{\rho }}$$

where *ρ* is fluid density, *A* is the cross-sectional surface of the pipeline and *K_∞_* is the flow coefficient in the given conditions of calibration (undisturbed flow in conditions of fully developed velocity profile). The value of this coefficient is relative mainly to the cross-section of the probe, location of pressure-tapping holes and the ratio of the dimension, which is characteristic, of the design of a probe to the diameter of a pipeline. In this case, given the relatively small diameter of the pipeline, the probe can cause additional flow resistance, which is reflected in a decrease in flow coefficient K. The K can be treated here as a parameter that is measured or calculated in specified flow condition in order to calculating level of measurement error during tests.

Within a low range of flow rates, this value is also relative to the Reynolds number and increases with values of Re described in Refs [15,16]. In industrial applications, we often encounter probes of various cross-sections, which is known to contribute to variations in the flow coefficient proposed in Refs [17,18,19]. In studies in recent years, attempts have been made to design probes with adequate metrological parameters, as described in Ref [20]. In addition, such probe designs need to be able to operate fault free in various conditions, including vibrations and stress as a result of fluid flow at various velocities. For the case where the fluid flow has a high velocity, we need to consider how the compressibility of the fluid has an effect on the value of K. This effect is accounted for in the form of a multiplier. Its value is relative to the Mach number, which is applied to account for the specific flow conditions shown in Ref [21,22,23] reported studies that numerically modeled the velocity and pressure fields for flow around the flow-averaging Pitot probe. The determination of the velocity profiles and pressures in the vicinity of a probe and inside it based on numerical studies can provide a better insight into the physics of the examined phenomena. Such research can offer information that is valuable for designers of new probes and can provide guidelines for their use. In case of a distortion in the velocity region, numerical studies offer insight into an assessment of the effect of flow distortion on registrations performed with a probe. Such studies can help explain the type of the phenomenon with a considerable influence on variations in the registration according to a flowmeter. They can also provide valuable information on the location of the probe that can help reduce the additional measurement error resulting from the installation of a probe.

**Figure 2:**
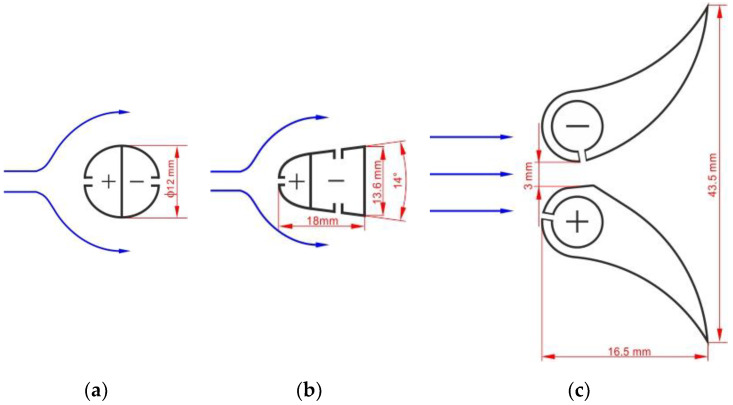
Averaging cross-sections of the Pitot tube. (**a**) circular, (**b**) streamlined, (**c**) two profile.

The present study and reports analyzed for the purposes shown in Refs [12,24,25] it was applied flowmeters with three types of cross-sections of flow averaging tubes (Figure 2). The probes are identified in accordance with their descriptions relating to cross-section. Probe (a) is below an example of a common design with a circular cross-section. For probes (b) and (c), the details of design can be found in Refs [22,23].

Probe in Figure 2a is a typical cylindrical probe with impulse holes (four each) on the inflow and outflow side, respectively. Probe in Figure 2b is a streamlined probe with a monolithic structure, with pre-drilled pressure averaging chambers. There are *p*^+^ holes on the front surface and *p*^−^, on both side surfaces. Probe in Figure 2c consists of two separate profiles. One of them has a *p*^+^ chamber and the other one has a *p*^−^ chamber. The *p*^+^ holes are located in the inflow part of the probe and the *p*^−^ holes in the second probe, between the profiles.

### 2.2. Test Stand

**Figure 3:**
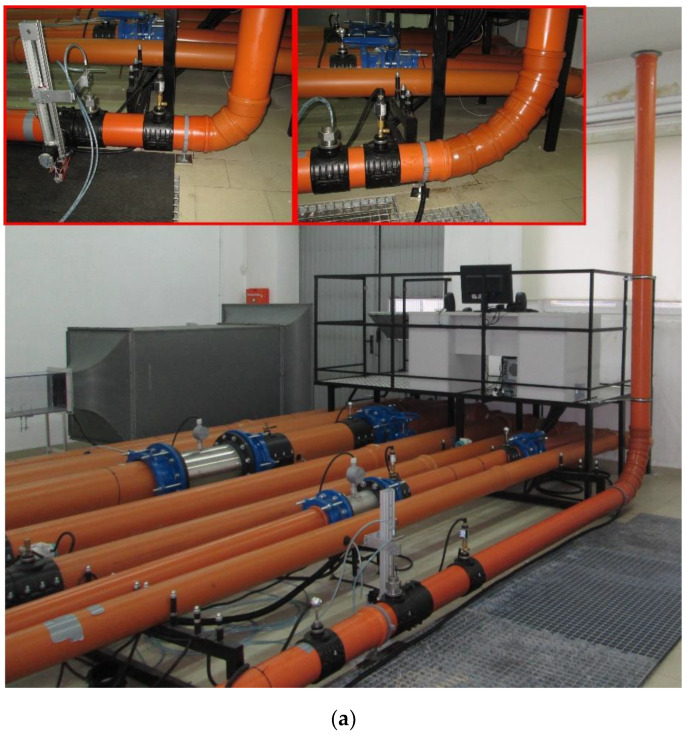

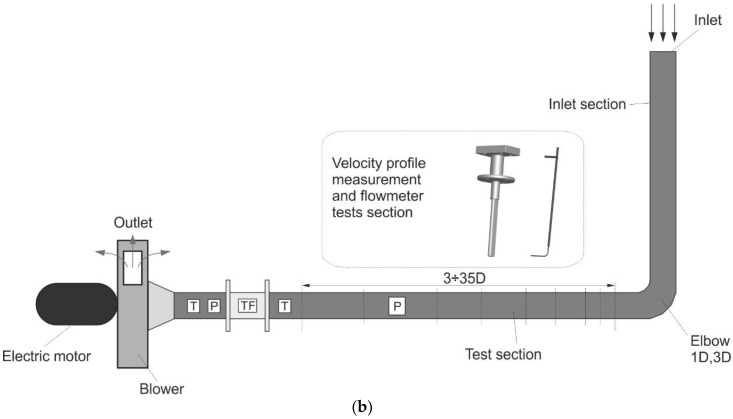
Experimental test stands. (**a**) general view with exemplary elbow obstacles, (**b**) schematic view: P—absolute pressure measurement, T—temperature measurement, TF—turbine flowmeter.

The research was conducted in a pipeline with an internal diameter of 152 mm. An image and a diagram of the experimental setup are presented in Figure 3a, b, respectively. The experimental setup comprised a system of pipelines with diameters ranging from DN 110 to DN 400 (DN—nominal diameter of pipeline), with two including precision turbine flowmeters with an additional measurement error of less than 0.5% applied for reference purposes, as described in Refs [12,20]. The setup also includes a wind tunnel and a top-of-the-range system for data registration, visualization and storage based on the LabView environment described in Ref [26]. The test stand was used to investigate flowmeters, with a particular emphasis on the assessment of the effect of flow disturbance in the velocity profile. The use of the high-quality measurement equipment enabled the determination of the total systematic error of the flow coefficient K with regard to the measured pressures and air temperatures for a constant uncertainty in the flow rate. This uncertainty was found to be in the range of ±0.71%.

The details of flow obstructions are summarized in Section 3.

**Figure 4:**
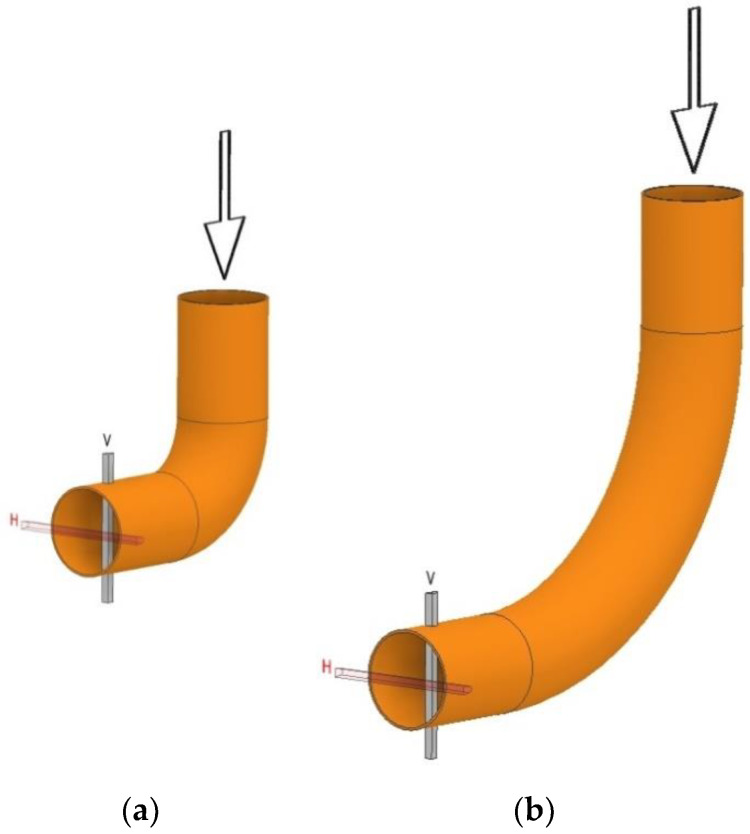
Single-elbow flow system of different curvatures (arrows mark the direction of flow) (**a**) R_c_ = 1D, (**b**) R_c_ = 3D.

The examined flowmeters were located at different distances from the obstruction in two mutually perpendicular planes, as schematically marked in Figure 4.

The process of determining the value of δ (systematic error) for the probes, for which the measurements resulting from the installation of elbows in various planes and elbows with specific radii of curvature equal to 1D and 3D, were compared with some values for the K coefficient reported in Refs [12,24,25].

### 2.3. Mathematical Model of Transient Flow

**Figure 5:**
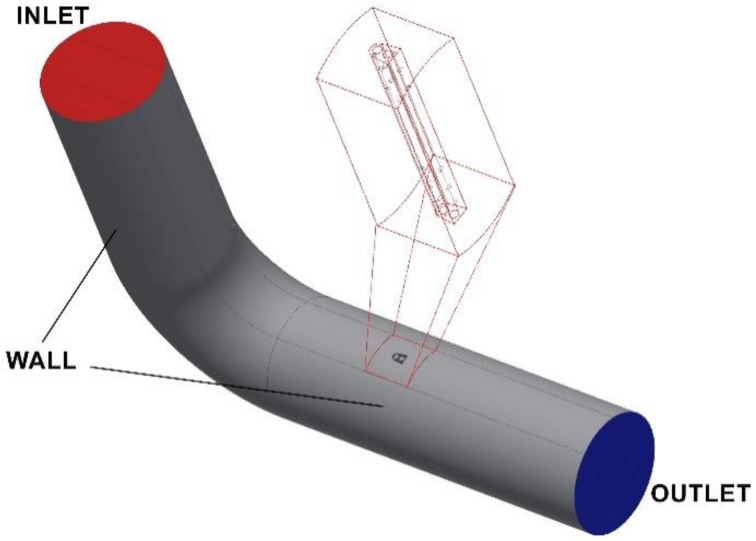
Boundary conditions for the analyzed flow system, with inlet and outlet sections and the enlarged part of the analyzed probe shortened (for better visualization).

The analysis of turbulent pipe flow downstream of an elbow for different curvature ratios was provided in Ref [27]. The numerical analysis involved an isothermal, turbulent flow assuming air as an incompressible fluid at mean velocities of 10 m/s, 18 m/s and 26 m/s through a 90-degree elbow with curvature radii 1D and 3D, as well as a streamlined probe located downstream of this system (i.e., in the direction of oncoming flow). The study was concerned with variable velocity profiles in the selected cross-section of the pipe downstream of the elbow, and in the vicinity of the probe. In addition, the analysis involved flow through the bore holes and inside the averaging chambers. Due to the characteristics of the phenomena, the issue was considered to be transient and three-dimensional (3D) (Figure 5).

Turbulent fluid flow for general incompressible and compressible fluid flows can be described by the equations of motion described in Ref [28]:

(2) $$\frac{\partial \left(\rho U_{i}\right)}{\partial t}+\frac{\partial \left(\rho U_{i}U_{j}\right)}{\partial x_{j}}=-\frac{\partial p}{\partial x_{i}}+\frac{\partial }{\partial x_{i}}\left[\mu_{ef}\left(\frac{\partial U_{i}}{\partial x_{j}}+\frac{\partial U_{j}}{\partial x_{i}}\right)\right]$$

and the equation of continuity

(3) $$\frac{\partial \rho }{\partial t}+\frac{\partial }{\partial x_{j}}\left(\rho U_{j}\right)=0$$

In the above equations, *U_i_* is a component of the velocity vector in directions *x*, *y* and *z*, *p* is pressure, *ρ* is fluid density and *µ_ef_* is effective viscosity as a sum of molecular, *µ* and turbulent, *µ_t_*, viscosities, i.e.,

(4) $$\mu_{ef}=\mu +\mu_{t}$$

The kinetic energy of turbulence k and its dissipation ε (or specific dissipation rate ε of turbulent kinetic energy in case of the k-ω model) are determined from the following transport equations:

(5) $$\frac{\partial }{\partial t}\left(\rho k\right)+\frac{\partial }{\partial x_{i}}\left(\rho kU_{i}\right)=\frac{\partial }{\partial x_{j}}\left(\Gamma_{k}\frac{\partial k}{\partial x_{j}}\right)+G_{k}-Y_{k}+S_{k}$$

(6) $$\frac{\partial }{\partial t}\left(\rho \omega \right)+\frac{\partial }{\partial x_{i}}\left(\rho \omega U_{i}\right)=\frac{\partial }{\partial x_{j}}\left(\Gamma_{\omega }\frac{\partial \omega }{\partial x_{j}}\right)+G_{\omega }-Y_{\omega }+S_{\omega }$$

where *G_k_* represents the generation of *k* and *G_ω_*, the generations of *ω*. *Γ_k_* and *Γ_ω_* represent the effective diffusivities of *k* and *ω,* respectively. In the above equations, *Y_k_* and *Y_ω_* represent the dissipations of *k* and *ω* due to turbulence *S_k_* and *S_ω_* are the source terms. The effective diffusivities *Γ_k_* and *Γ_ω_* are calculated as described below, according to Ref [28]:

(7) $$\Gamma_{k}=\mu +\frac{\mu_{t}}{\sigma_{k}}$$

(8) $$\Gamma_{\omega }=\mu +\frac{\mu_{t}}{\sigma_{\omega }}$$

where $\sigma_{k}\;\mathrm{and}\;\sigma_{\omega }$ are the turbulent Prandtl numbers for *k* (turbulent kinetic energy) and *ω* (specific dissipation rate), respectively. The turbulent viscosity *µ_t_* is calculated from

(9) $$\mu_{t}=\rho C_{\mu }\frac{k}{\omega },$$

where *C_µ_* is a model constant equal to 0.09.

Details of the particular quantities applied in Equations (9)–(11) as well as the constants used are given in Ref [28].

### 2.4. Mathematical and Numerical Models of Flow around the Probe

**Figure 6:**
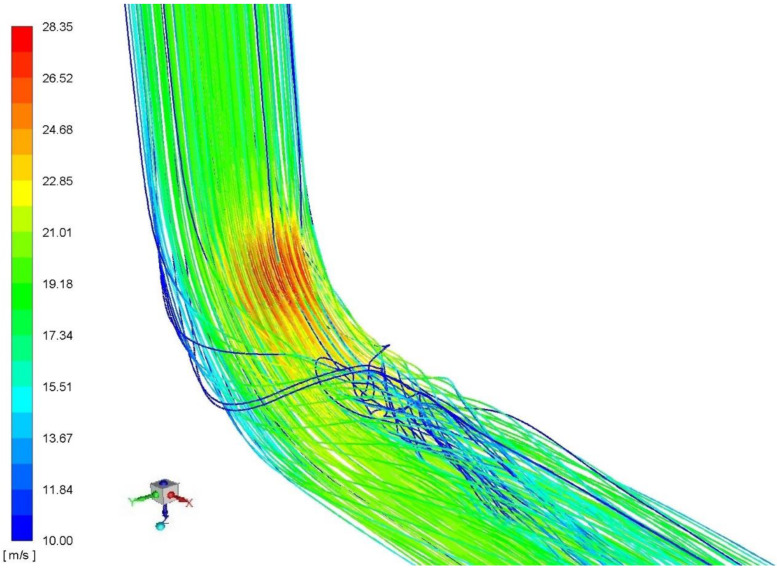
Unsteady flow through an elbow with a radius of curvature R_c_ = 1D at mean velocity 18 m/s.

The computational region was applied in accordance with Figure 6 and the boundary conditions were:

INLET (at the inlet)–*k* = *1.22 m*^2^/*s*^2^, *ω* = *189.4 1/s, U_x_* = *18.0 m/s, U_y_* = 0; *U_z_* = 0
OUTLET (at the outlet)-$\frac{\partial U_{x}}{\partial x}=0;\frac{\partial U_{y}}{\partial x}=0;\frac{\partial U_{Z}}{\partial x}=0;\frac{\partial k}{\partial x}=0;\frac{\partial \omega }{\partial x}=0$
WALL (along all walls of the pipelines and the probe)-*U_x_* = 0; *U_y_* = 0; *U_z_* = 0

Basic parameters was set as:

Scheme—SIMPLE

Spatial discretization:

Gradient—least square cell-based
Pressure—second-order
Momentum—second-order upwind
Turbulent kinetic energy—second-order upwind,
Specific dissipation rate—second-order upwind
Transient Formulation—second-order implicit

The following criteria was set for the transient computations:

max iteration per time step-20
continuity–10^−5^
x-velocity–10^−6^
y-velocity–10^−6^
z-velocity–10^−6^
k–10^−6^
omega–10^−6^

The geometry of this system is shown in Figure 5, where the simulations apply to a system comprising an elbow with a radius of curvature R_c_ = 1 (Figure 4a). The authors here present simulations of an elbow with the smallest radius of curvature, as this case was considered the extreme in terms of flow metrology. In such a system, we can observe phenomena characteristic of a result of flow distortion resulting from variations in the direction of flow and the effect of the walls of the elbow with a small radius of curvature on the fluid. We can thus conclude that the observed phenomena had non-stationary characteristics, combined with a region corresponding to intensive turbulence downstream of an elbow, and along a certain distance down it in the vicinity of the top wall section shown in Figure 6.

The discrete region was developed on the basis of hexahedra, tetrahedra and wedge elements as described in Ref [28]. Mesh tests were performed to select an optimum number and density of the mesh elements in regions with considerable gradients of pressure and velocity, and accounting for the boundary layer inside the pipeline—on the exterior part of the probe in the flowmeters as well as inside the bore holes and averaging chambers. This was achieved by ensuring that the values *y^+^* were suited to the applied turbulence model, in accordance with Ref [28]. In addition, the time step was adapted to achieve the optimum values (Δt = 10^−5^ s at w = 18 m/s) by applying a computing machine unit comprising 56 state-of-the-art processors and 96 GB RAM. The usual duration of computations was around 120 h. In order to check mesh convergence, the tests were carried out to find their optimal dense and distribution. Tests were performed for average parameters: velocity w = 18 m/s and distance x/D = 10. The resulting meshes are presented in Table 1.

## 3. Results and Discussion

The results of the research are elaborated by comparing the volume flow rate measured by the tested flowmeter with the registration performed with a reference flowmeter. Throughout the measurements performed using these flowmeters, the value of the flow coefficient *K_∞_* was assumed to be a constant value, which corresponded to the fully developed velocity profile for a given Re number. The difference in flow rates was expressed in relation to the reference value *q_vref_* and was expressed in percentage. It was assumed to represent the additional error with a systematic character and was marked by index *δ*, i.e.,

(10) $$\delta =\frac{\left(q_{v}-q_{v}{}_{ref}\right)}{q_{v}{}_{ref}}\cdot 100\%$$

By applying the above formula and the relation in (1), following transformations, we obtain

(11) $$\delta =\frac{\left(K_{\infty }-K\right)}{K}\;\cdot 100\%$$

where *K_∞_* is the value of the flow coefficient for the fully developed flow for a given Re number. Flow coefficient K is formed by a coefficient resulting from Equation (1), for the case when the flowmeter is found downstream of an obstruction. We are familiar with the actual flow *q_vref_* and the value Δ*p* measured in such conditions. The additional measurement error resulting from the disturbance in the velocity profile was determined for three averaging probes located at distances equal to 3D, 4D, 5D, 7D, 8D, 9D, 10D, 12D, 14D, 18D and 22D behind the obstacle as shown in Figure 3b.

**Figure 7:**
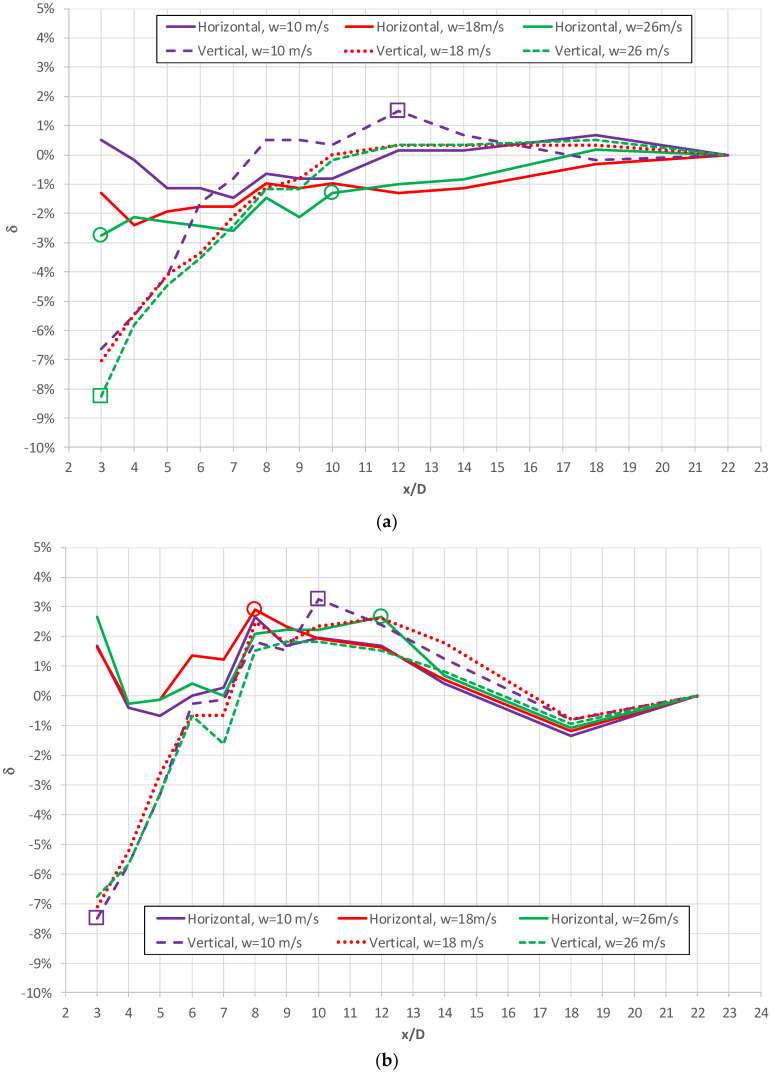
Results of additional error δ for various distances downstream of an elbow with radius of curvature R_c_ = 1D (additional symbol means maximum additional error—square for vertical position and circular for horizontal position of probe, respectively) for cases of: (**a**) circular and (**b**) streamlined probes (based on data from [24]).

As an example, Figure 7a,b shows the results of measurements performed with regard to the probes with circular (Figure 7a) and streamlined (Figure 7b) cross-sections installed at various distances downstream of a pipe elbow with a radius of curvature *R_c_* = 1D. This distance is expressed in relation to the pipeline diameter D. In addition, Figure 7a,b contains circular (horizontal) and square (vertical) symbols that mark the maximum values of systematic error resulting from the distortion in the velocity profile. The values of the absolute systematic error summarized for all alternatives are summarized in Table 1. In Figure 7a,b, as well as in Table 1, the cases for which the measurements of the flow rates are presented for the range of distances x (linear parameter parallel to flow direction) equal to 3 ≤ x/D < 10 and x/D ≥ 10 down from the obstacle were distinguished.

In addition, Figure 7a,b show information on the mean velocities of flow (10 m/s, 18 m/s and 26 m/s), for which charts were developed. Using these curves for the case of the probe with a circular cross-section (Figure 7a), we can conclude a direct correlation between the registered values for δ corresponding to higher flow velocities (18 m/s and 26 m/s) for the vertical orientation of the probe (V) and a slightly lower similarity for the horizontal installation (H) of the probe for the same velocities. This can be explained by the characteristics of the stream distortion forming directly downstream of an obstacle formed by an elbow with a small curvature R_c_ = 1D, as described in Refs [12,27,29]. For the case of the streamlined probe (Figure 7a), a considerable similarity of characteristics was recorded for both the horizontal (H) and the vertical (V) probe locations at lower flow velocities (10 and 18 m/s).

The results show that in case of a circular probe, its horizontal location resulted in a considerably smaller systematic error in measurements that can be attributed to flow distortion, in comparison with the vertical installation of the probe. A similar conclusion can be made with regard to the streamlined probe for the location of the flowmeter, which was smaller than x/D = 8 from the pipe elbow.

**Figure 8:**
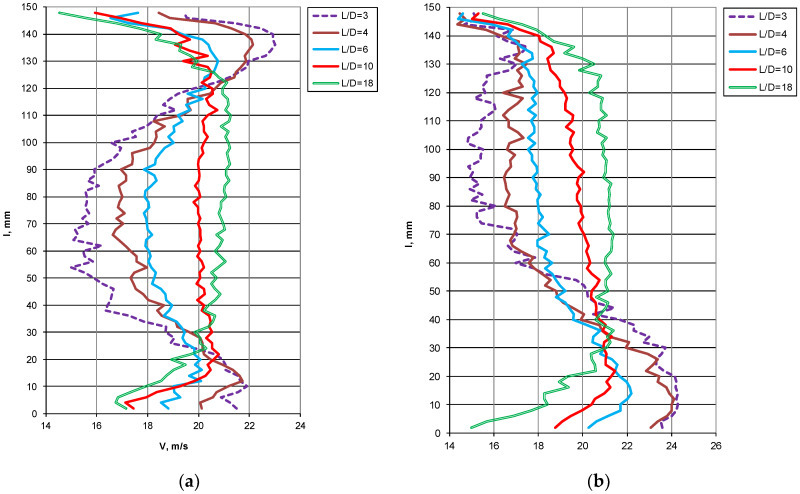
Velocity profile behind an Rc = 1D elbow at mean velocity w = 18 m/s. (**a**) horizontal position, (**b**) vertical position.

Velocity profile recovery behind the elbow in vertical and horizontal position significantly differs from each other (Figure 8). Practically, it means that different total pressure inflows to averaging chambers through individual impulse holes. Velocity profile in horizontal plane has more symmetric shape than in vertical plane. That is the main advantage of locating probe in this position and rather avoiding vertical one.

For practical application, the results of the study are elaborated in the form of a table (Table 2) and the maximum value of δ is given for each of the analyzed probes and types of obstruction (Figure 4), as well as for the values of the mean air velocities of 10, 18 and 26 m/s. (Re = 1.02 − 2.65∙10^5^). The values of *δ* are given for the location of the probe at a distance smaller than x/D=10 from the obstruction as well as in the range x/D = 10 to 22. The information in Table 1 offers some important considerations in probe selection and provides important clues for its potential location. Moreover, the locations marked in Table 1 denote points that offer a systematic error |δ|<2%. This value adds to uncertainty provided by the probe manufacturers, and offers an insight regarding, e.g., the potential applications of the probe as an element responsible for the control of the flow rate. Note that the use of a standard orifice leads to a higher degree of flow resistance, and, thus, the flow is affected. Consequently, longer sections of the pipeline are required (in the range of several to tens of the length of x/D) upstream of the probe, as shown in Refs [11,12,13]. In particular, this effect is prominent for cases when the beta ratio $\beta =\frac{d}{D}$ (d—diameter of orifice opening, D—internal diameter of a pipeline) of the orifice plates is large.

The influence of liquid flow on a probe in the flowmeter is a complex issue. Such phenomena pose difficulties, both in experimental research presented in Refs. [21,25] and in the numerical modeling shown in Refs [22,23,30]. We should emphasize that, in this study, the major task is the analysis of a system in conditions where flow around a probe is modeled, and flow in both the bore holes and the flow-averaging chambers needs to be considered. As we have mentioned above, the standard k-ω model forms the turbulence model that can represent the phenomena occurring in this system. This model performs well in the analysis of flow featuring vortices and recirculation phenomena as well as intensive pressure and velocity gradients. Owing to a lack of detailed experimental data, numerical simulations can be verified globally by developing a dependence to relate the volumetric flow rate *q_v_* to differential pressure Δ*p* in the tapping holes of the flow-averaging chambers:

(12) $$K=\frac{q_{v}}{A}\sqrt{\frac{\rho }{2\Delta p}}$$

The impact of flow disturbance can be expressed through the ratio *K*/*K_∞_*_._

**Figure 9:**
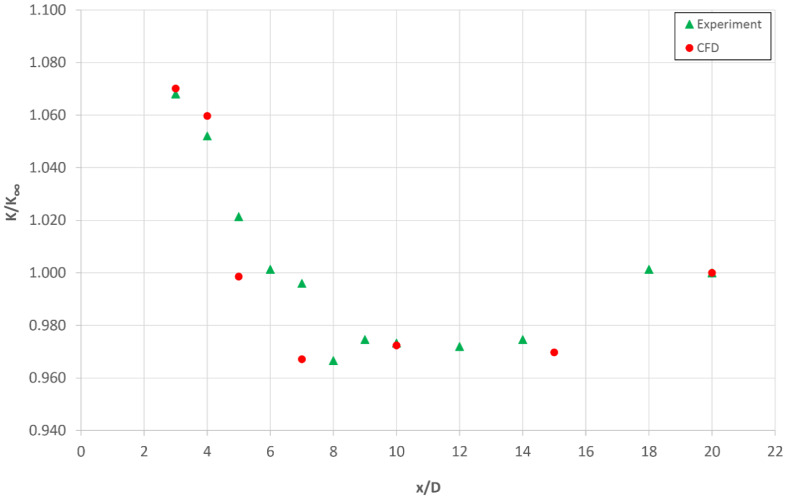
Relative value of the flow coefficient (*K/K*_∞_) for the case of distortion formed by a single elbow 1-D and vertical position of the streamlined probe at mean velocity w = 18 m/s.

Figure 9 shows examples of experimental and numerical data with regard to flow downstream of an elbow with a radius of curvature of R_c_ = 1D (mean flow velocity w = 18 m/s). These results exhibit a considerable degree of quantitative validity. This justifies the application of the mathematical model and numerical simulations in studies on flow inside a flowmeter and offers ground for the statement of metrological conclusions.

The mathematical model and numerical simulations were applied to analyze the phenomena occurring around the probe. This provided information needed to establish a reply to the question about the influence of the distortion of the velocity profile on the measurements of the averaged value of flow rate, and the dominant effect of some flow phenomena on the results.

This issue was analyzed using the effects of the distortion in the velocity profile resulting from the installation of a pipe elbow (R_c_ = 1D) on the registrations performed by the probe with a streamlined cross-section. The numerical study was compared with the first of the cases analyzed in Table 1—that is, for the vertical installation of the probe, as the most difficult case of the analyzed metrological problem.

**Figure 10:**
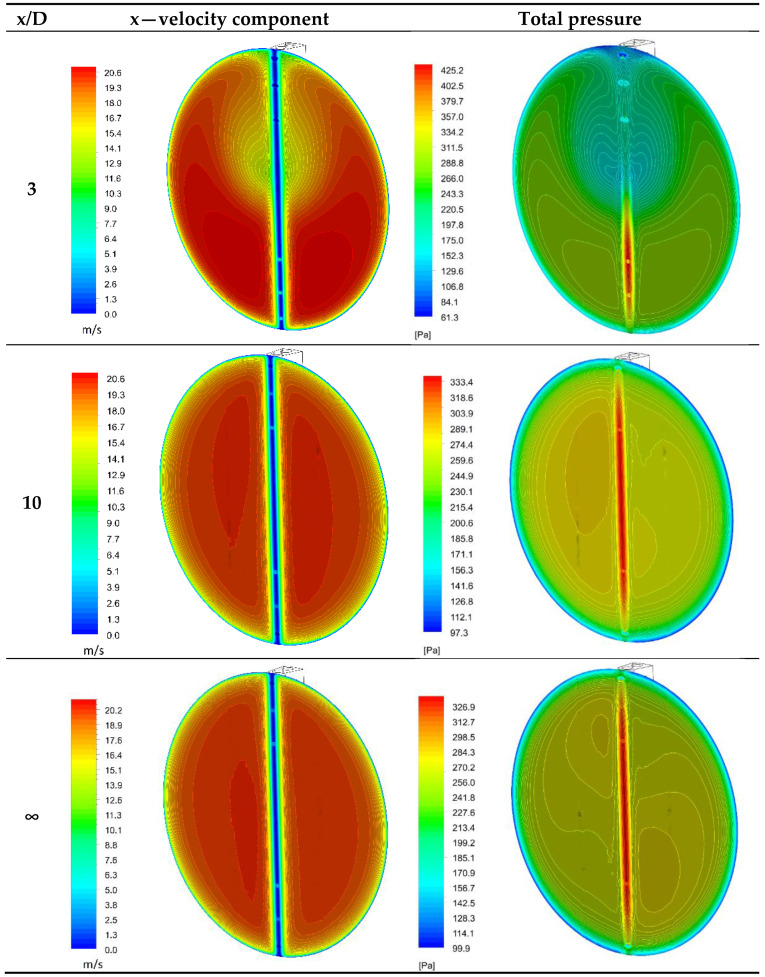
Distributions of axial velocity and total pressure in the tangential plane upstream of the streamlined probe (for mean velocity w = 18 m/s).

The results of the study are presented in Figure 10. They show that the considerable distortion resulting from the installation of the pipe elbow significantly affected the distribution of total pressure on the surface of the probe. For the distance x/D = 3, these distributions had a considerable degree of asymmetry in the analyzed cross-sections. This is reflected by the differentiation in the values of pressure and velocity in the planes corresponding to the locations of the bore holes. The intensive effect of flow on the probe occurred virtually along its bottom part located closer to the outer part of the elbow bend. The dynamic differential pressure in the symmetrical bore holes of the second and fourth chambers p^+^ was 86.7 Pa for a mean flow velocity of w = 18 m/s. Such conditions occur particularly within ratios of x/D in the range of three to seven. However, at x/D = 10, the velocity profile was sufficiently developed, and only slightly deviated from the undisturbed flow (denoted by “∞”).

**Figure 11:**
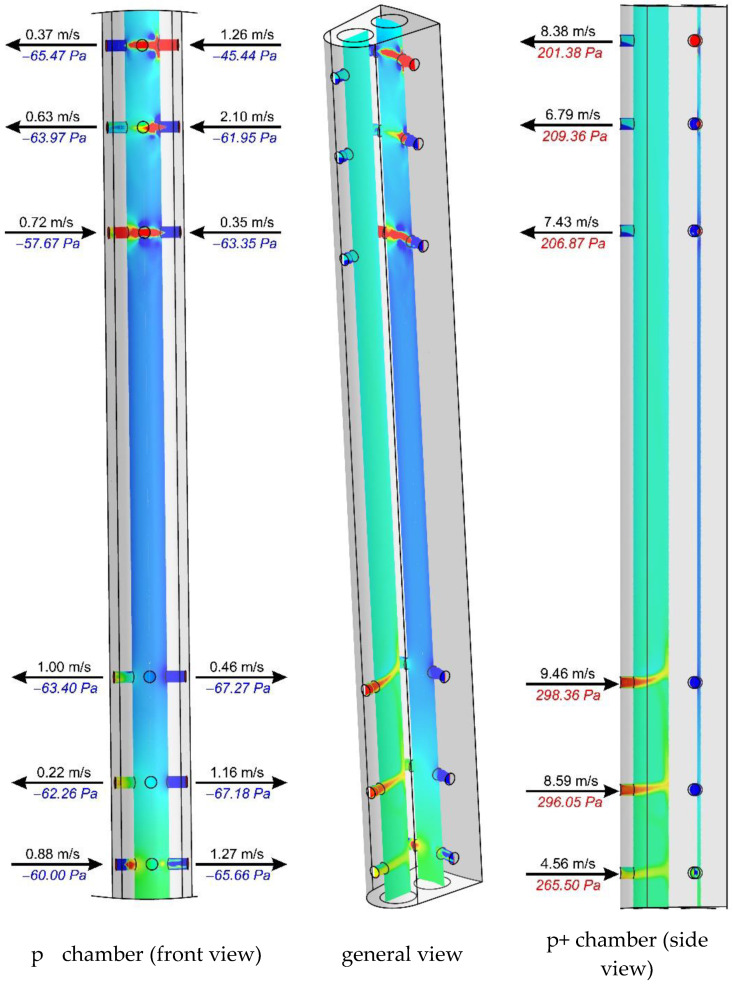
Fluid velocity and total pressure in planes of bore holes downstream of an elbow (x/D = 3) with radius of curvature R_c_ = 1D.

**Figure 12:**
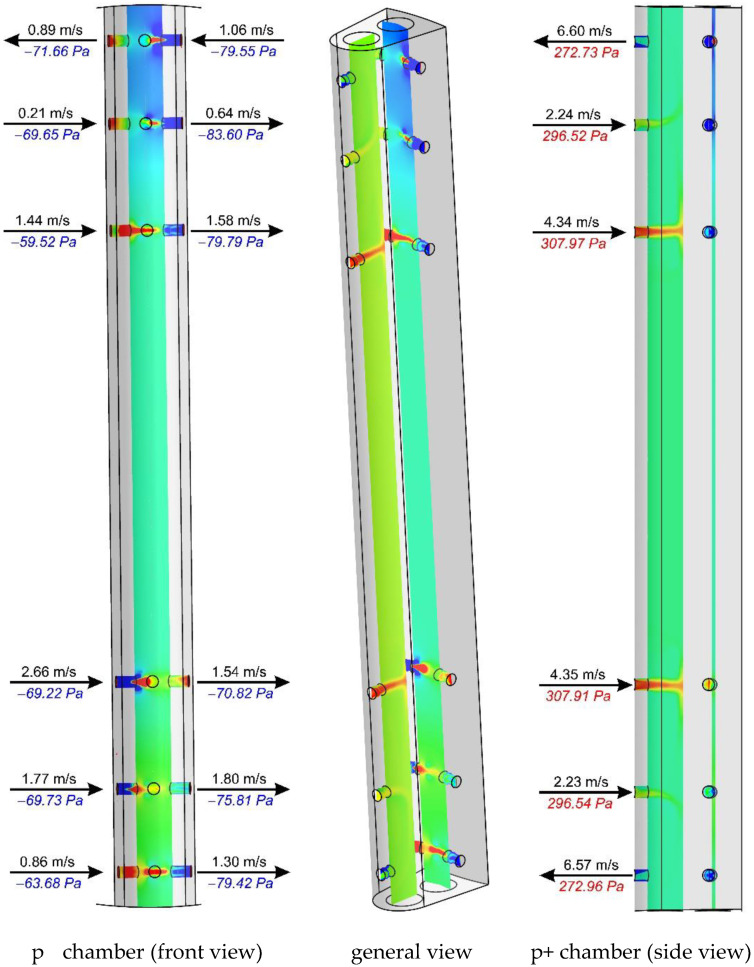
Fluid velocity and total pressure in planes of the bore holes downstream of an elbow (x/D = 10) with curvature R_c_ = 1D.

**Figure 13:**
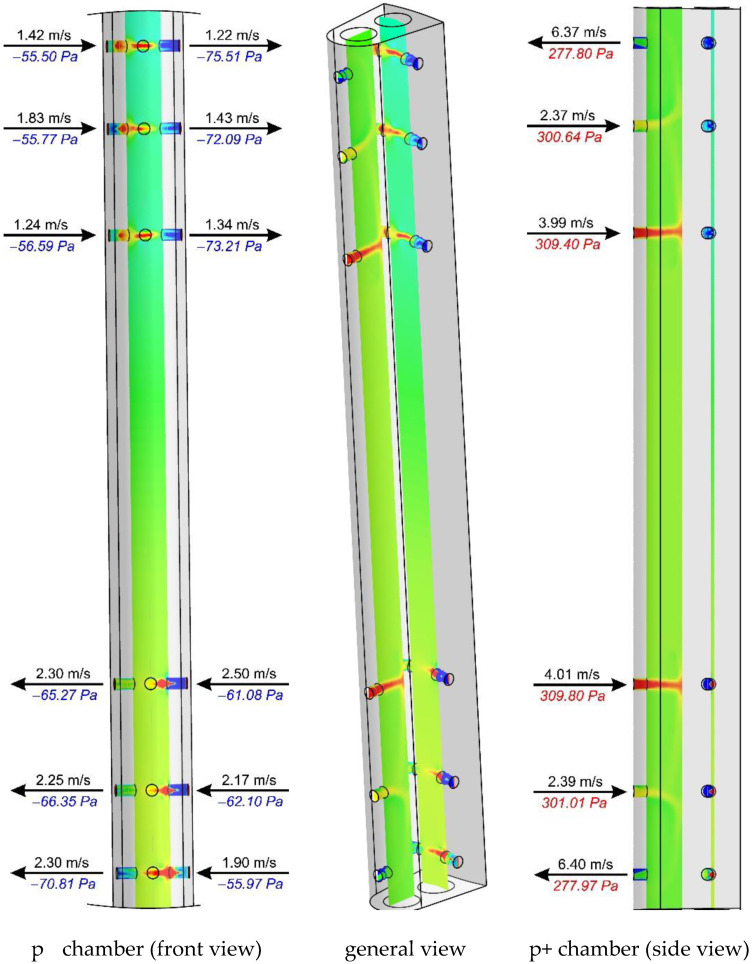
Fluid velocity and total pressure in planes of the bore holes downstream of an elbow (x/D = ∞) with curvature R_c_ = 1D.

Figure 11, Figure 12 and Figure 13 show the distributions of total pressures in the averaging chambers as well as the component of the velocity parallel to the axes of the bore holes at a given instant in time. The results are presented for three distances downstream of an elbow, just as for the case in Figure 10. For clarity of presentation, each case is presented at the same angle (side view—p^+^ chamber, general view and a front view—p^−^ chamber). This analysis provides complementary results to the distributions of the total pressure and velocity recorded in the tangential plane to the stagnation line of the flow-averaging probe. In further figures, i.e., Figure 11, Figure 12 and Figure 13, we clearly see an asymmetry of flow through a negative pressure-averaging chamber, which confirms the need to perform a numerical analysis for a complete 3D view in a non-stationary form. The initial stationary computations performed by the application of system symmetry did not offer satisfactory conclusions.

In spite of the various directions and velocities in the planes at the inlet of the bore holes, the authors noted slight differences in total pressure for x/D = 10 and ∞ (Figure 10), which is further reflected in the small differences in the distribution of pressure inside the averaging chambers at these distances, as visually represented by Figure 12 and Figure 13. Examples of probe locations at 3-D and 10 D are characterized by the distinct direction of flow in various bore holes, both with regard to the p^+^ as well as p^−^ chambers. This is associated with the different distributions of velocities and pressures in the area of the streamlined flow around the probe.

**Figure 14:**
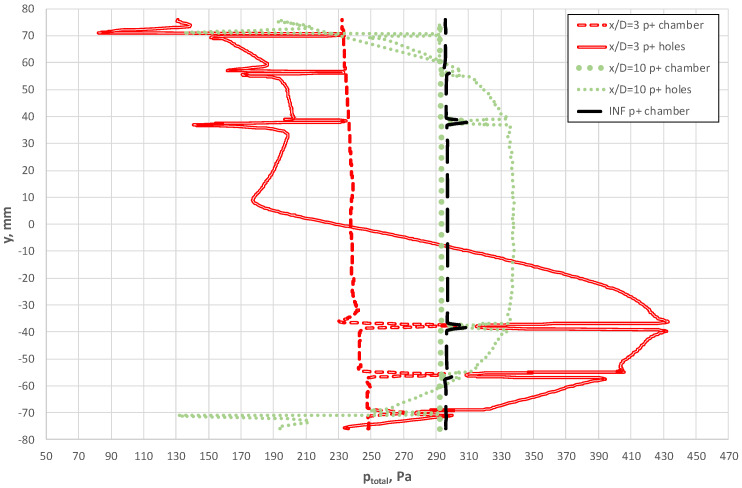
Total pressure along the axis of the p^+^ chamber and along the line that combines the p^+^ holes on the side of the probe.

The numerical studies prove the occurrence of aerodynamic phenomena, the effect of which is relevant in the examined case and can be shown to play a role in other probes and their locations. The higher value of the K-factor, for the case of the streamlined probe located in the vertical position at distance x/D = 3 downstream of the elbow (*K*/*K_∞_* = 1.07), findings from a lower value of Δ*p* caused by non-axial fluid flow around the probe. This flow has a dominant character around the circumference and the bottom section of the elbow. This is practically demonstrated by the pressure distribution in the front section of the probe (Figure 14). This effect leads to a complete alteration in the direction and flow rate through bore holes, in comparison with the case where flow with an undistorted velocity profile is investigated (Figure 13). This results in the additional occurrence of considerable total pressure gradients in the vicinity of the bore holes. The characteristics of these variations (Figure 14) are attributable to the direction of the flow in the bore hole, i.e., whether it occurs inside or outside the probe (Figure 11 and Figure 13). Liquid flow through the holes, and the relatively considerable ratio of dynamic pressure in the total pressure, is demonstrated by peaks of the total pressure recorded around the holes, both for the case of the stagnation line as well as in the axes of the averaging chambers.

**Figure 15:**
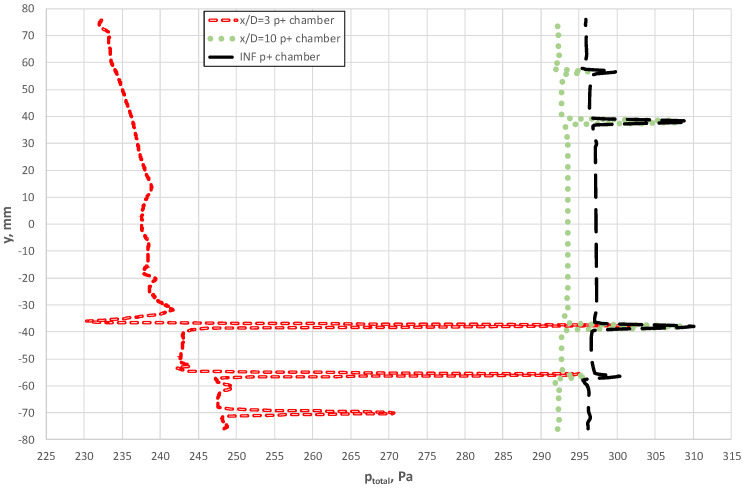
Total pressure along the axis of the p^+^ chamber at various distances between the probe and the elbow.

In Figure 15, we see how the total pressure varied along the line combining p^+^ holes upstream side of the probe when it was located at a distance 3D from the elbow. This figure shows a clear asymmetry of flow upstream for the averaging probe. For the case of the probe located at a distance 10 D, the range of variable total pressures along the line that combines the bore holes was similar to the case x/D = ∞ (INF), as illustrated in Figure 15. Variations in total pressure around the bore holes close to the axis of the pipeline were also characteristics for this case. Such a distribution of the pressure corresponded to the case where flow with the fully developed velocity profile occurred along the probe surface.

**Figure 16:**
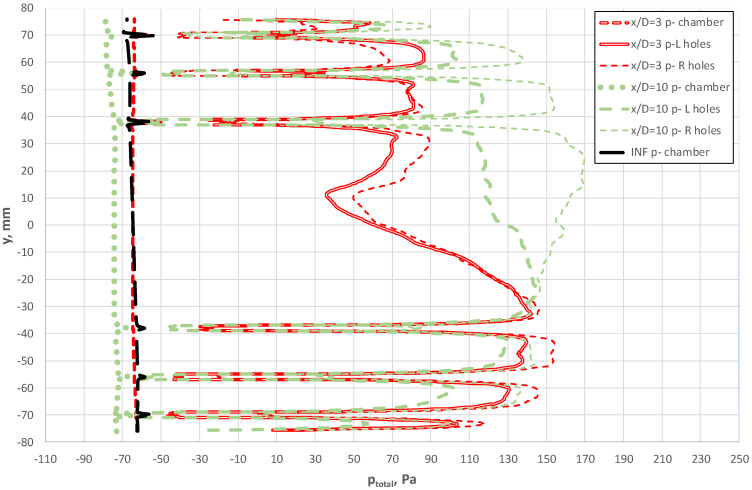
Total pressure along the axis of the p^−^ chamber and along the lines combining the p^−^ hole on the external probe surface.

The distribution of total pressures along the negative pressure-averaging chambers is summarized in Figure 16. In addition, this figure shows the pressures in the cross-sections of the bore hole, developed independently for each of the sides (left and right) of the flow-averaging probe at a given instant in time. The curve demonstrates a qualitative similarity of pressure distribution in the averaging chambers for all three cases. In terms of the values of total pressure along the line that combines the bore holes outside the probe, we note differences between cases for x/D = 3 and x/D = 10, as well as between distributions of total pressure on the right and left side of the probe. The difference between the distributions on the two sides of the probe demonstrate the clearly unsteady and 3D characteristics of liquid flow. It is noteworthy that these differences were greater for the location of the probe x/D = 10.

**Figure 17:**
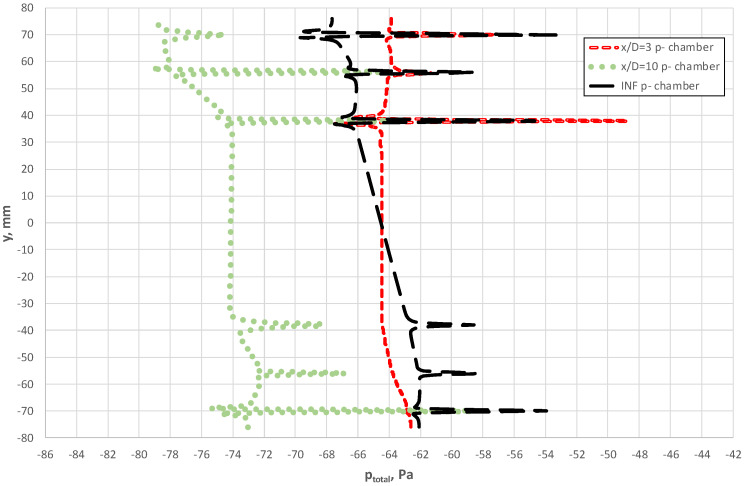
Total pressure along the axis of the p^−^ chamber at various distances between the probe and the elbow.

**Figure 18:**
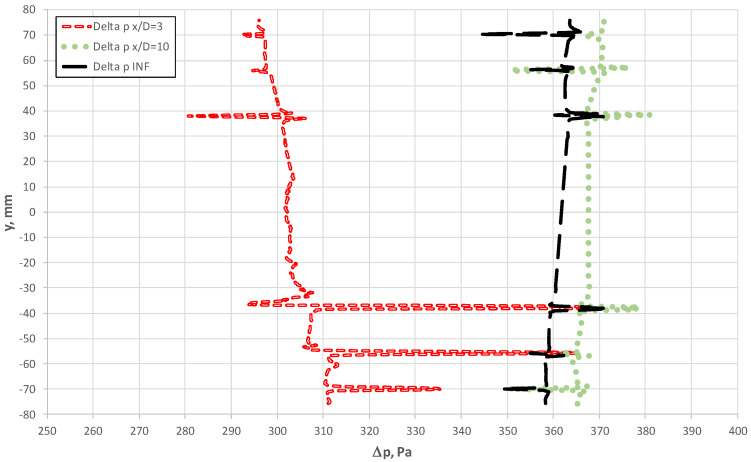
Differential pressure along axes of the flow-averaging chambers (Δp = p^+^ − p^−^).

Figure 17 shows how total pressure in the chambers also varies as a function of probe length. Nevertheless, the absolute range of these changes is considerably smaller in comparison with p^+^. At the same time, we note from Figure 15, Figure 16, Figure 17 and Figure 18 that the chambers were capable of effectively fulfilling their role of flow averaging on condition of an adequate selection of the diameter of the averaging probe chamber d_k_ in relation to that of the bore hole d_o_ (d_k_/d_0_ ≈3). The chambers could average pressure for the case when the probe was located close downstream of the obstruction, despite large gradients (peaks) of pressure in the vicinity of the tapping holes. This effect led to greater measurement uncertainty when the velocity profile varied locally. For example, this was the case for the distance x/D = 3 downstream of the elbow.

Figure 18 illustrates the value of the differential pressure between the chambers as a function of probe length. In the investigated case, the value and distribution of the pressures for x/D = 10 only slightly deviated from the fully developed flow. This resulted in a relatively low value of additional measurement error (2.33%) for the distance x/D > 10. For the case where the probe was installed at x/D = 3, we see the local effect of flow through the bore holes on the values of Δp on the bottom of the probe. The distribution of the variables and volumetric rate in the bore holes (Figure 13) consequently led to a lower pressure recorded in the p^+^ chamber (Figure 14), which yielded lower values of Δ*p*.

The distributions of velocities and pressures around the probe forms a complex issue and is relative to the cross-section of the probe, flow obstruction and the distance between this element and the probe. All these factors need to be investigated separately. This is confirmed by the experimental research. Nevertheless, the results summarized in Table 1 can be applied to engineering.

The numerical analysis here provides grounds for the assessment of additional measurement error resulting from a flow disturbance. This study offered a way to determine the distance between the probe and the obstruction for the case where this systematic error is within admissible levels in conditions specified by exploitation. Numerical modeling can also provide guidelines to modify the design of the probe, including the number and location of the bore holes. Such modifications can help improve and adapt the metrological characteristics of flowmeters to the particular conditions of exploitation.

## 4. Conclusions

This research offers the following conclusions:

(a) The results of detailed studies summarized in Table 1 provide information on the distance between the probe and the obstruction and offer insight into its installation in a way that can lead to a reduction in additional measurement error. As shown in Table 1, the results of this systematic error can assume considerable values with a different sign. An additional error in the range 2–3% can sometimes be considered acceptable for technological installations and automatic regulation systems.
(b) In some investigated cases, the standard k-ω turbulence model when applied provided good quantitative conformity between the calculated and the measured differential pressures inside the averaging chambers.
(c) In some cases, mathematical modeling and numerical simulations can provide useful insights into assessing this additional measurement error. Most importantly, information regarding phenomena of flow around a probe and the effect of the distortion of a velocity field can be obtained.
(d) The numerical simulations conducted on the streamlined probe located downstream of a single elbow provide grounds for the analysis of the distribution of pressures and velocities inside and around flow-averaging chambers. Despite the considerable variations in total pressure along the surface of the probe, the applied diameters of the bore holes and averaging chambers offer a successful averaging of pressures in the chambers. In the investigated case of calculations, the distribution of pressures in the p^+^ chamber assume a state of balance. In general, we can conclude that in this case, the value of the averaged pressure in the p^+^ chamber had a decisive effect on the additional measurement error. Consequently, we see that the distance between the pipe elbow and the place of installation of the probe affected the distribution of pressures and the value Δp registered between the pressure-averaging chambers.
(e) Mathematical modeling can provide valuable insights into details of the installation and further adaptations of the probe design to improve the quality of measurements. A way to limit the additional measurement error will be the location of the holes in the probe that will allow the best possible averaging of the deformed velocity profile. This requires numerical calculations, analysis of the velocity profile at the location of the probe and selection of points better representing the average velocity in cross-section.

## Figures and Tables

**Table 1:** Parameters of analyzed mesh.

Mesh	Super Coarse	Very Coarse	Coarse	Little Coarse	Fine	Very Fine	Super Fine
Overall dimension increase	130%	125%	120%	115%	110%	105%	100%
Error ref. to Δp of superfine mesh	−4.1%	−42.4%	0.6%	0.3%	0.3%	−40.1%	0%
Number of elements	4 837 549	5 257 604	5 771 527	7 982 892	8 204 543	8 610 560	9 676 486
Aspect Ratio	1–40.155	1–43.9	1–44.9	1–46.1	1–47.1	1–47.7	1–45.4
Skewness range	0–0.883	0–0.893	0–0.846	0–0.896	0–0.869	0–0.897	0–0.833
Orthogonal Quality range	0.116–1	0.106–1	0.154–1	0.104–1	0.131–1	0.103–1	0.133–1
Max element length, mm	2.17	2.12	2.08	1.80	1.89	1.83	1.68

**Table 2:** Maximum values of additional measurement error resulting from disturbance (single elbows of different radii) of the velocity profile (**V**—vertical position, **H**—horizontal installation of the probe).

Flowmeter Cross−Section	Radius of Single Elbow, R_c_	Orientation of Probe in Relation to Curvature Plane	Maximum Measurement Error at the Distance	
			<10D,%	≥10D,%
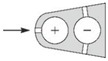	1D	V	−7.49	3.26
		H	2.90	2.66
	3D	V	2.62	1.48
		H	−1.28	−1.02
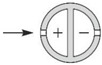	1D	V	−8.26	1.51
		H	−2.76	−1.32
	3D	V	−4.15	0.42
		H	−1.99	−0.49
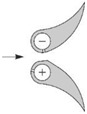	1D	V	−3.24	−2.88
		H	2.02	1.38
	3D	V	−3.07	−1.76
		H	−1.20	0.43

## Nomenclature

*C_d_ (-)*	Discharge coefficient
*q_v_* (*m*^3^/*s*)	Volumetric flow rate
*K (-)*	Flow coefficient (empirical)
*K_∞_ (-)*	Flow coefficient referred to undisturbed flow
*A* (*m*^2^)	Cross-sectional area of pipeline
Δ*p (Pa)*	Differential pressure
*ρ* (*kg/m*^3^)	Fluid density
*DN (mm)*	Nominal diameter of pipeline
*D (mm)*	Diameter of pipeline
*U_i_ (m/s)*	Components of velocity vector along directions *x*, *y*, *z,* respectively
*p (Pa)*	Absolute pressure
*µ_ef_ (Pa⋅s)*	Effective viscosity
*µ_t_ (Pa⋅s)*	Turbulent viscosity (eddy viscosity)
*µ (Pa⋅s)*	Molecular viscosity
*k* (*m*^2^/*s*^2^)	Turbulent kinetic energy
*ω (1/s)*	Specific dissipation rate
*w (m/s)*	Mean velocity of fluid
*G_k_* (*kg/m∙s*^3^)	Generation of turbulent kinetic energy k
*G_ω_* (*kg/m^3^∙s*^2^)	Generation of specific dissipation rate ω
*Γ_k_ (kg/m∙s)*	Effective diffusivity of k
*Γ_ω_ (kg/m∙s)*	Effective diffusivity of ω
*Y_k_* (*kg/m∙s*^3^)	Dissipation of k due to turbulence
*Y_ω_* (*kg/m^3^∙s*^2^)	Dissipation of ω due to turbulence
*S_k_* (*kg/m∙s*^3^)	Source term referring to k
*S_ω_* (*kg/m*^3^*∙s*^2^)	Source term referring to ω
*$\sigma_{k}$ (-)*	Turbulent Prandtl number for k
*$\sigma_{\omega }$(-)*	Turbulent Prandtl number for ω
*C_μ_ (-)*	Turbulence model, constant equal to 0.09
*R_c_ (mm)*	Elbow radius of curvature with reference to pipeline diameter D
*Re (-)*	Reynolds number
*q_vref_* (*m*^3^/*s*)	Reference volumetric flow rate
*x (m)*	Longitudinal coordinate in flow direction
*p* ^+^ *(Pa)*	Positive pressure
*p* ^−^ *(Pa)*	Negative pressure
*d_k_ (mm)*	Average chamber probe diameter
*d*_0_ *(mm)*	Probe impulse hole diameter
*δ (%)*	Systematic error in fluid flow measurement
*y* ^+^ *(-)*	Boundary layer thickness (solution-dependent parameter)

## References

[1] Lupeau A., Platet B., Gajan P., Strzelecki A., Escande J., Couput J.P. (2007). Influence of the presence of an upstream annular liquid film on the wet gas flow measured by a Venturi in a downward vertical configuration. Flow Meas. Instrum..

[2] Li Y., Jun W., Geng Y. (2009). Study on wet gas online flow rate measurement based on dual slotted orifice plate. Flow Meas. Instrum..

[3] Adefila K., Yan Y., Sun L., Wang T. (2017). Flow measurement of wet CO_2_ using an averaging pitot tube and Coriolis mass flowmeters. Int. J. Greenh. Gas. Con..

[4] Berrebi J., Martinsson P.-E., Willatzen M., Delsing J. (2004). Ultrasonic flow metering errors due to pulsating flow. Flow Meas. Instrum..

[5] (2016). ISO 5167-5. Measurement of Fluid Flow by Means of Pressure Differential Devices Inserted in Circular Cross-Section Conduits Running Full—Part 5: Cone Meters.

[6] Weissenbrunner A., Fiebach A., Schmelter S., Bär M., Thamsen P.U., Lederer T. (2016). Simulation-based determination of systematic errors of ﬂow meters due to uncertain inﬂow conditions. Flow Meas. Instrum..

[7] Singh R.K., Singh S.N., Seshadri V. (2009). Study on the effect of vertex angle and upstream swirl on the performance characteristics of cone flowmeter using CFD. Flow Meas. Instrum..

[8] Singh R.K., Singhb S.N., Seshadri V. (2010). CFD prediction of the effects of the upstream elbow fittings on the performance of cone flowmeters. Flow Meas. Instrum..

[9] He D., Bai B. (2017). Gas-liquid two phase flow with high GVF through a horizontal V-Cone throttle device. Int. J. Multiph. Flow.

[10] He D., Chen S., Bai B. (2018). Experiment and Numerical Simulation on Gas-Liquid Annular Flow through a Cone Sensor. Sensors.

[11] Chmielniak T., Kotowicz J., Węcel D. (2008). Experimental and numerical investigations of the averaging Pitot tube and analysis of installation effects on the flow coefficient. Flow Meas. Instrum..

[12] Kabaciński M., Pochwała S., Pospolita J. (2012). Influence of Typical Flow Disturbing Elements on the Flow Rate in Selected Averaging Pitot Tubes. TASK Q..

[13] Spitzer D.W. (1991). Flow Measurement: Practical guides for measurement and control. Isa Res. Triangle Park..

[14] Turkowski M., Szufleński P. (2013). New criteria for the experimental validation of CFD simulations. Flow Meas. Instrum..

[15] Vinod V., Chandran T., Padmakumar G., Rajan K.K. (2012). Calibration of an averaging pitot tube by numerical simulations. Flow Meas. Instrum..

[16] (2018). ALLPRONIX Data Sheet Flow Meters. https://allpronix.com/product-category/flow-meters/.

[17] (2018). Fayin Instruments Data sheet Flow Meters. http://www.fayin.com.tw..

[18] (2018). Meriam’s Accutube Flow Sensors Data Sheet. http://www.testequipmentdepot.com/meriam/pdf/accutube_series.pdf..

[19] (2018). Preso Flow Metering Equipment, Data Sheet. http://www.mesure.com/docs/Ellipse.pdf.

[20] Kabaciński M., Pospolita J. (2011). Experimental research into a new design of flow—Averaging tube. Flow Meas. Instrum..

[21] Kotzé R., Wiklund J., Haldenwang R., Fester V. (2011). Measurement and analysis of flow behavior in complex geometries using the Ultrasonic Velocity Profiling (UVP) technique. Flow Meas. Instrum..

[22] Kabaciński M., Pospolita J. (2008). Numerical and experimental research on new cross-sections of averaging Pitot tubes. Flow Meas. Instrum..

[23] Dobrowolski B., Kabaciński M., Pospolita J. (2005). A mathematical model of the self—Averaging Pitot tube A mathematical model of a flow sensor. Flow Meas. Instrum..

[24] Kabaciński M. (2016). Influence of disturbed flow after an elbow on metrological properties of a Flow Averaging Tube. Meas. Autom. Monit..

[25] Kabaciński M., Pochwała S. (2011). The impact of velocity profile deformation on flow coefficient value for flowmeter with tube averaging dynamic pressure. Meas. Autom. Control.

[26] Kring J., Travis J. (2006). LabVIEW for Everyone: Graphical Programming Made Easy and Fun.

[27] Chowdhury R.R., Alam M.M. (2016). Turbulent flow analysis on bend and downstream of the bend for different curvature ratio. AIP Conf. Proc..

[28] (2018). ANSYS/FLUENT v.19.1 Documentation. https://www.pharea-software.com/content/uploads/2018/05/Release_Notes_191.pdf.

[29] Kim J., Yadav M., Kim S. (2014). Characteristics of secondary flow induced by 90-degree elbow in turbulent pipe flow. Eng. Appl. Comp. Fluid.

[30] Kabaciński M., Pospolita J. (2006). Applicability of the chosen turbulence models in numerical investigations of flows around a body with stream separation. Inz. Chem. Procesowa.

